# Effects of sacubitril/valsartan in ESRD patients undergoing hemodialysis with HFpEF

**DOI:** 10.3389/fcvm.2022.955780

**Published:** 2022-11-09

**Authors:** Yanhong Guo, Mingjing Ren, Tingting Wang, Yulin Wang, Tian Pu, Xiaodan Li, Lu Yu, Liuwei Wang, Peipei Liu, Lin Tang

**Affiliations:** ^1^Department of Nephropathy, The First Affiliated Hospital of Zhengzhou University, Zhengzhou, China; ^2^Department of Gastroenterology, Wenxian People’s Hospital, Jiaozuo, China; ^3^Department of Gastroenterology, The First Affiliated Hospital of Zhengzhou University, Zhengzhou, China; ^4^Clinical Systems Biology Laboratories, The First Affiliated Hospital of Zhengzhou University, Zhengzhou, China

**Keywords:** heart failure with preserved ejection fraction, hemodialysis, sacubitril/valsartan, left ventricle dysfunction, pulmonary hypertension

## Abstract

**Introduction:**

Heart failure with preserved ejection fraction (HFpEF), which is a common co-morbidity in patients with maintenance hemodialysis (MHD), results in substantial mortality and morbidity. However, there are still no effective therapeutic drugs available for HFpEF currently. Sacubitril/valsartan has been shown to significantly improve clinical outcomes and reverse myocardial remodeling among patients with heart failure with reduced ejection fraction (HFrEF). The effect of sacubitril/valsartan in MHD patients with HFpEF remains unclear. Our study was designed to assess the efficacy and safety of sacubitril/valsartan in MHD patients with HFpEF.

**Methods:**

A total of 247 MHD patients with HFpEF treated with sacubitril/valsartan were included in this retrospective study. Patients were followed up regularly after medication treatment. The alterations in clinical, biochemical, and echocardiographic parameters before and after taking sacubitril/valsartan were collected. In addition, the safety of the sacubitril/valsartan treatment was also assessed. Among those 247 patients with MHD, 211 patients were already in treatment with angiotensin converting enzyme inhibitors (ACEi)/angiotensin receptor blockers (ARBs) before being treated with sacubitril/valsartan. We also performed an analysis to compare the differences between the 211 patients who had previously received ACEi/ARB treatment and the 36 patients who were sacubitril/valsartan naive.

**Results:**

Among those 247 patients with MHD, compared with baseline levels, systolic blood pressure (BP) (149.7 ± 23.6 vs. 137.2 ± 21.0 mmHg, *P* < 0.001), diastolic BP (90.2 ± 16.1 vs. 84.5 ± 14.1 mmHg, *P* < 0.001), heart rate (83.5 ± 12.5 vs. 80.0 ± 8.7 bpm, *P* < 0.001), N-terminal B-type natriuretic peptide precursor (NT-proBNP) [29125.0 (11474.5, 68532.0) vs. 12561.3 (4035.0, 37575.0) pg/ml, *P* < 0.001], and cardiac troponin I [0.044 (0.025, 0.078) vs. 0.0370 (0.020, 0.064) μg/L, *P* = 0.009] were markedly decreased after treatment with sacubitril/valsartan. New York Heart Association (NYHA) functional class showed a notable trend of improvement after 3–12 months of follow-up. Echocardiographic parameters including left ventricular posterior wall thickness (LVPWT) (11.8 ± 2.0 vs. 10.8 ± 1.9 mm, *P* < 0.001), intraventricular septal thickness in diastole (11.8 ± 2.0 vs. 11.2 ± 2.0 mm, *P* < 0.001), left ventricular end-diastolic diameter (53.8 ± 6.9 vs. 51.2 ± 7.1 mm, *P* < 0.001), left atrial diameter (LAD) (40.5 ± 6.2 vs. 37.2 ± 7.2 mm, *P* < 0.001), left ventricular end-diastolic volume (LVEDV) [143.0 (111.5, 174.0) vs. 130.0 (105.0, 163.0) ml, *P* < 0.001], left ventricular end-systolic volume (LVESV) [57.0 (43.0, 82.5) vs. 48.0 (38.0, 74.0) ml, *P* < 0.001], and pulmonary arterial systolic pressure [39.0 (30.5, 50.0) vs. 28.0 (21.0, 37.5) mmHg, *P* < 0.001] were significantly reduced after initiating the treatment of sacubitril/valsartan. The parameters of left ventricular diastolic function including E/A ratio [0.8 (0.7, 1.3) vs. 0.9 (0.8, 1.3), *P* = 0.008], maximal tricuspid regurgitation velocity [2.7 (2.5, 3.2) vs. 2.4 (2.0, 2.8) m/s, *P* < 0.001], septal e’wave velocity (8.0 ± 0.6 vs. 8.2 ± 0.5 cm/s, *P* = 0.001), lateral e’ wave velocity (9.9 ± 0.8 vs. 10.2 ± 0.7 cm/s, *P* < 0.001), E/e’ [8.3 (6.4, 11.8) vs. 7.2 (6.1, 8.9), *P* < 0.001], and left atrial volume index (37.9 ± 4.2 vs. 36.4 ± 4.1 ml/m^2^, *P* < 0.001) were significantly improved by sacubitril/valsartan. Among 211 patients who were already in treatment with ACEi/ARB and 36 patients who were sacubitril/valsartan naive, the improvement of cardiac function demonstrated by clinical outcomes and echocardiographic parameters were similar to the previous one of the 247 MHD patients with HFpEF. During the follow-up, none of the patients showed severe adverse drug reactions.

**Conclusion:**

Our study suggested that sacubitril/valsartan treatment in MHD patients with HFpEF was effective and safe.

## Introduction

Epidemiological studies showed that there were 697.5 million cases of chronic kidney disease (CKD) worldwide in 2017, with a global prevalence of 9.1% (1). Between 1990 and 2017, the global mortality rate from CKD increased by 41.5% in all age groups worldwide (1). Hemodialysis is an effective renal replacement therapy for patients with end-stage renal disease (ESRD). At present, approximately 89% of patients with dialysis receive hemodialysis worldwide (2). Studies have shown that heart failure is the common co-morbidity of patients with ESRD undergoing maintenance hemodialysis (MHD), with an incidence of up to 40% in this patient population (3). Meanwhile, heart failure is also the leading cause of death in patients with MHD (4). Once heart failure is present in patients with ESRD undergoing hemodialysis, the survival level is significantly worse (4, 5). Among patients with dialysis, the most common form of heart failure is heart failure with preserved ejection fraction (HFpEF), which is present in approximately 20% of patients with MHD (6). A prospective cohort study showed that 81% of patients have HFpEF among MHD patients with heart failure (7).

Available evidence confirms that among MHD patients with HFpEF, the main pharmacological treatment is aimed at the management of volume overload, hypertension, and myocardial ischemia (8). There is currently little evidence that beta-blockers, angiotensin converting enzyme inhibitors (ACEi), angiotensin receptor blockers (ARBs), spironolactone, and ivabradine are beneficial in MHD patients with HFpEF (9–13). However, these drugs are usually used in the management of associated co-morbidities, such as hypertension and coronary artery disease. Recently published evidence shows that sacubitril/valsartan improves heart failure symptoms in patients with HFpEF (14). However, the effect of sacubitril/valsartan in MHD patients with HFpEF remains unclear. Thus, this study aimed to investigate the effects of sacubitril/valsartan on MHD patients with HFpEF.

## Materials and methods

### Study design and patients

**FIGURE 1:**
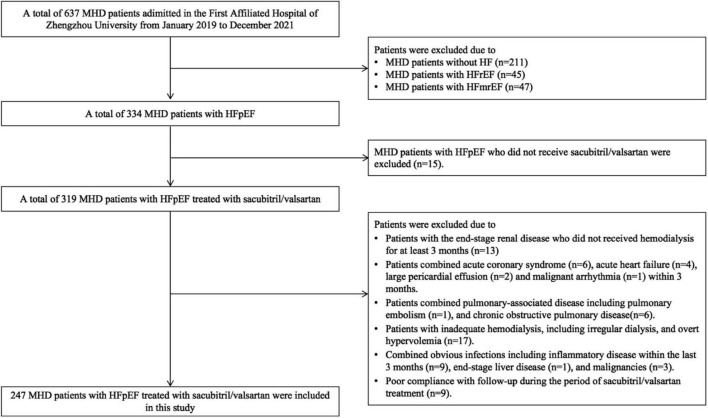
Study flowchart.

This was a retrospective study. A total of 247 MHD patients with HFpEF administered in the First Affiliated Hospital of Zhengzhou University between January 2019 and December 2021 with the following criteria were included in this retrospective study: (1) All patients received hemodialysis treatment for at least 3 months and were aged ≥ 18 years at baseline; (2) All patients were diagnosed as CKD according to KDIGO guidelines (15); (3) All patients were diagnosed as HFpEF; (4) All patients had complete clinical data; and (5) All MHD patients with HFpEF were prescribed sacubitril/valsartan. During the follow-up, the concomitant therapy and dialysis regimen of those patients were stable. The exclusion criteria were as follows: (1) combined acute coronary syndrome, acute heart failure, malignant arrhythmia, pericardial disease, and unstable hemodynamics patients; (2) combined pulmonary-associated disease including asthma attack, pulmonary embolism, or chronic obstructive pulmonary disease; (3) inadequate hemodialysis, including irregular dialysis, overt hypervolemia, symptomatic hypotension, or systolic blood pressure < 100 mmHg at screening; (4) combined obvious infections including inflammatory disease within the last 3 months, end-stage liver disease, and malignancies; and (5) poor compliance with follow-up during the period of sacubitril/valsartan treatment (Figure 1).

The diagnosis of HFpEF in patients without ESRD relies on history, physical examination, laboratory tests including N-terminal B-type natriuretic peptide precursor (NT-proBNP), and echocardiographic examination. However, the level of NT-proBNP is affected by the reduction of renal excretion, so NT-proBNP was elevated in kidney dysfunction even in the absence of HF. At present, there is no critical value for NT-proBNP in the diagnosis of HFpEF among the dialysis population (16). However, the level of NT-proBNP may still reflect the presence and severity of heart failure. Therefore, for patients with MHD, the diagnosis of HFpEF is still based on symptoms/signs and evidence of cardiac structural or functional abnormalities according to the diagnostic criteria from the European Society of Cardiology (17, 18). Patients were diagnosed with pulmonary hypertension (PAH) based on echocardiography- estimated systolic pulmonary artery pressure ≥ 35 mmHg. Hyperkalaemia was defined as a serum potassium concentration > 5.5 mmol/L. The H2FPEF score includes echocardiographic and clinical variables, and the European Society of Cardiology HFA-PEFF score was calculated as a sum of points in functional, morphological, and biomarker domains (19, 20).

All patients were given conventional treatments for HFpEF, including hemodialysis, reducing volume load, and blood pressure control. On the basis of usual treatment, sacubitril/valsartan was administered as alternatives to ACEi or ARB after consultation with the cardiologist with a starting dose of 50 mg bid, which was increased by one dose every 2 weeks until the maximum tolerated dose for blood pressure or the target dose (200 mg bid). If the predialysis systolic blood pressure was less than 110 mmHg, the sacubitril/valsartan dose did not need to be increased. No patients discontinued the drug during the follow-up. All patients were required to undergo serum potassium tests once in half a month until stabilization.

Between January 2019 and December 2021, there were 211 MHD patients without heart failure. Echocardiographic data and NT-proBNP data of these 211 MHD patients without HF and the comparison to 247 MHD patients with HFpEF were shown in Supplementary File. Among those 247 MHD patients with HFpEF, 211 patients were already in treatment with ACEi/ARB before being treated with sacubitril/valsartan. We also analyzed the differences in the clinical data before and after the treatment of sacubitril/valsartan among those 211 patients who were already treated with ACEi/ARB and 36 patients who were sacubitril/valsartan naive. To analyze whether the results would be affected by the use of ACEi/ARB or not, we provided an analysis to compare the differences in the clinical data of the two groups. The study protocol was approved by the local ethics committee, and all patients provided written informed consent.

### Clinical data collection

Prior to sacubitril/valsartan administration, general clinical parameters including gender, age, height, body weight, duration of hemodialysis, primary renal disease, blood pressure, and heart rate were collected. After 3–12 months of treatment with sacubitril/valsartan, cardiac structure and function were assessed by echocardiography and New York Heart Association (NYHA) functional class. Echocardiography was performed by two independent and experienced sonographers. Echocardiographic indicators included right ventricle diastolic diameter (RVDd), left ventricular posterior wall thickness (LVPWT), ascending aorta (AAO), E/A ratio, right atrial diameter (RAD), left ventricular end-diastolic volume (LVEDV), left ventricular end-systolic volume (LVESV), interventricular septum diastolic thickness (IVSd), left ventricular ejection fraction (LVEF), maximal tricuspid regurgitation velocity (TRVmax), Septal e’wave velocity, Lateral e’ wave velocity, E/e’, left atrial volume index (LAVI), left ventricular end-diastolic diameter (LVDd), pulmonary artery systolic pressure (PASP), and left atrial diameter (LAD). In addition, we also collected the expression levels of other cardiac biomarkers, including creatine kinase MB (CK-MB), cardiac troponin I (TNI), and NT-proBNP. We observed the occurrence of adverse events such as hyperkalemia, hypotension, cough, and angioedema at the same time.

In addition to parameters related to cardiac function, laboratory variables were also collected. Blood urea nitrogen (BUN), uric acid, serum calcium, triglycerides (TG), and serum albumin were measured by colorimetry using Cobas c 701 (Roche, Basel, Switzerland). Serum creatinine was measured by enzymatic methods using Cobas c 701 (Roche, Basel, Switzerland). Lipid metabolism-related indicators such as total cholesterol (TC), high-density lipoprotein cholesterol (HDL-C), and low-density lipoprotein cholesterol (LDL-C) were measured by enzymatic colorimetry using Cobas c 701 (Roche, Basel, Switzerland). NT-proBNP was measured by electrochemiluminescence using Cobas e 602 (Roche, Basel, Switzerland). Intact parathyroid hormone (PTH) was measured by electrochemiluminescence using Cobas e 801 (Roche, Basel, Switzerland).

### Statistical analysis

Patient characteristics were described by mean ± standard deviation, medians, interquartile ranges, and percentages according to the data type. The percentage changes (%) of the clinical data were calculated as follows: percentage change = (Data at the end of follow-up—Data at baseline) × 100/Data at baseline. If the data are in accordance with normal distribution, paired sample *t*-test was performed for comparison between the two groups. If the data did not conform to the normal distribution, the Wilcoxon matched-pair signed-rank (two samples) test was used for comparison between the two groups. McNemar’s test was used to assess differences between categorical variables. All of these data were analyzed using the SPSS 23.0 software package and *p*-values were calculated as two-sided. Statistical significance was set at *P* < 0.05.

## Results

### Baseline characteristics of 247 patients with maintenance hemodialysis

Between January 2019 and December 2021, 247 patients with MHD who were treated with sacubitril/valsartan were recruited to participate in this retrospective study. The baseline demographic data of those patients are shown in Table 1. The average age of those 247 patients with MHD was 45.8 ± 13.7 years with a male/female ratio of 154/93. The mean BMI was 23.4 ± 4.3 kg/m^2^ and the mean duration of hemodialysis was 17.0 (4.3, 34.0) months. Among those patients, ESRD resulted from chronic glomerulonephritis (45.7%), diabetic kidney disease (31.6%), hypertensive nephropathy (9.3%), obstructive nephropathy (2.0%), autosomal dominant polycystic kidney disease (2.4%), Alport syndrome (0.8%), and others (10.1%). The median H2FPEF score of those patients was 2.0 (IQR: 1.0, 3.0), and the HFA-PEFF score was 5.1 ± 1.2 (Table 1).

**TABLE 1:** Baseline characteristics of patients undergoing hemodialysis with HFpEF initially presenting before sacubitril/valsartan treatment.

Variables	All patients (*n* = 247)	ACEi/ARB treated patients (*n* = 211)	Sacubitril/Valsartan naive patients (*n* = 36)
**Demographics**			
Gender (male/female)	154/93	138/73	16/20^a^
Age (year)	45.8 ± 13.7	45.4 ± 13.7	48.2 ± 13.4
BMI (kg/m^2^)	23.4 ± 4.3	24.1 ± 3.5	22.3 ± 3.2^a^
Duration of hemodialysis (months)	26.0 (14.0, 43.5)	24.0 (13.0, 36.5)	27.0 (14.5, 44.0)
**Causes of ESRD**			
Chronic glomerulonephritis, n (%)	113 (45.7%)	99 (46.9%)	14 (38.9%)
Diabetic kidney disease, n (%)	78 (31.6%)	69 (32.7%)	9 (25.0%)
Hypertensive nephropathy, n (%)	23 (9.3%)	18 (8.5%)	5 (13.9%)
Obstructive nephropathy, n (%)	5 (2.0%)	2 (0.9%)	3 (8.3%)
Polycystic kidney, n (%)	6 (2.4%)	1 (0.5%)	5 (13.9%)^a^
Alport syndrome, n (%)	2 (0.8%)	2 (0.9%)	0 (0.0%)
Others, n (%)	25 (10.1%)	20 (9.5%)	5 (13.9%)
**Co-morbidities**			
Hypertension, n (%)	247 (100.0%)	211 (100.0%)	36 (100.0%)
Diabetes, n (%)	89 (36.0%)	81 (38.4%)	8 (22.2%)
Dyslipidemia, n (%)	81 (32.3%)	69 (32.7%)	12 (33.3%)
Coronary artery disease, n (%)	21 (8.5%)	19 (9.0%)	2 (5.6%)
Atrial fibrillation, n (%)	10 (4.0%)	9 (4.3%)	1 (2.8%)
Prior stroke, n (%)	13 (5.2%)	11 (5.2%)	2 (5.6%)
Peripheral arterial disease, n (%)	3 (1.2%)	3 (1.4%)	0 (0.0%)
Obesity, n (%)	14 (5.6%)	14 (6.6%)	0 (0.0%)
**Medication use**			
Calcium channel blocker, n (%)	213 (86.2%)	186 (88.2%)	27 (75.0%)
β-Blocker, n (%)	185 (74.9%)	165 (78.2%)	20 (55.6%)^a^
Diuretics, n (%)	12 (4.9%)	10 (4.7%)	2 (5.6%)
ACE inhibitor or ARB, n (%)	211 (85.4%)	211 (100%)	0 (0.0%)
MRAs, n (%)	12 (4.9%)	9 (4.3%)	3 (8.3%)
α-Blocker, n (%)	161 (65.2%)	151 (71.6%)	10 (27.8%)^a^
H2FPEF score	2.0 (1.0, 3.0)	2.0 (1.0, 3.0)	2.0 (1.0, 2.0)
HFA-PEFF score	5.1 ± 1.2	5.1 ± 1.2	5.0 ± 1.3

Data presented as median (first-third interquartile range) or mean ± SD or number (percentage).

^*a*^Stands for p < 0.05 vs. ACEi/ARB treated patients.

HFpEF, Heart failure with preserved ejection fraction; BMI, body mass index; ESRD, end stage renal disease; ACEi, angiotensin-converting enzyme inhibitors; ARB, angiotensin II receptor blocker; MRAs, mineralocorticoid receptor antagonists; H2FPEF score = Heavy, 2 or more Hypertensive drugs, atrial Fibrillation, Pulmonary hypertension [pulmonary artery systolic pressure > 35 mm Hg], Elder age > 60, elevated Filling pressures [E/e’ > 9]; HFA-PEFF = Heart Failure Association diagnostic algorithm - Pre-test assessment, Echocardiography and Natriuretic Peptide Score, Functional testing, Final aetiology.

### Comparison of the clinic outcomes of 247 patients with maintenance hemodialysis before and after initiating sacubitril/valsartan

The follow-up period ranged from 3 to 12 months (median: 8.5 months). After 3–12 months of follow-up, compared to the baseline levels, systolic BP (149.7 ± 23.6 vs. 137.2 ± 21.0 mmHg, *P* < 0.001), diastolic BP (90.2 ± 16.1 vs. 84.5 ± 14.1 mmHg, *P* < 0.001), and heart rate (83.5 ± 12.5 vs. 80.0 ± 8.7 bpm, *P* < 0.001) were significantly lower than before. Moreover, the levels of NT-proBNP [29125.0 (11474.5, 68532.0) vs. 12561.3 (4035.0, 37575.0) pg/ml, *P* < 0.001] and TNI [0.044 (0.025, 0.078) vs. 0.370 (0.020, 0.064) μg/L, *P* = 0.009] were markedly reduced after the treatment with sacubitril/valsartan. At the end of the follow-up, the median percentage reduction of NT-proBNP level from baseline was –49.0% (interquartile range: –84.0 to 37.4%). No differences were found in other laboratory values including hemoglobin, TC, TG, HDL, LDL, urea, creatinine, uric acid, potassium, calcium, phosphorus, intact PTH, and albumin (Table 2).

**TABLE 2:** Comparisons of the characteristics of patients undergoing hemodialysis with HFpEF before and after initiating sacubitril/valsartan with observation period of 3–12 months.

Variables	Before sacubitril/valsartan	After sacubitril/valsartan	*P*-value
**Clinical parameters**			
SBP (mmHg)	149.7 ± 23.6	137.2 ± 21.0	<0.001
DBP (mmHg)	90.2 ± 16.1	84.5 ± 14.1	<0.001
Heart rate (bpm)	83.5 ± 12.5	80.0 ± 8.7	<0.001
**Laboratory values**			
Hemoglobin (g/L)	98.1 ± 22.9	101.5 ± 20.9	0.088
TC (mmol/L)	3.9 (3.3, 4.6)	4.0 (3.3, 4.7)	0.806
TG (mmol/L)	1.2 (0.8, 1.7)	1.1 (0.8, 1.6)	0.605
HDL-C (mmol/L)	2.5 (1.9, 3.1)	2.3 (1.7, 3.0)	0.273
LDL-C (mmol/L)	1.0 (0.8, 1.4)	1.5 (1.1, 2.0)	0.161
Urea (mmol/L)	27.2 (20.6, 32.8)	25.2 (19.1, 31.9)	0.409
Creatinine (μmol/L)	975.5 (811.0, 1187.1)	935.0 (726.2, 1150.5)	0.899
Uric acid (μmol/L)	386.0 (328.8, 466.0)	376.0 (302.5, 460.0)	0.533
Potassium (mmol/L)	4.4 ± 0.8	4.4 ± 0.8	0.279
Calcium (mmol/L)	2.2 (2.0, 2.3)	2.2 (2.1, 2.3)	0.127
Phosphorus (mmol/L)	2.0 (1.6, 2.4)	1.9 (1.5, 2.4)	0.547
Hyperkalemia [*n* (%)]	14 (5.7%)	20 (8.1%)	0.286
Intact PTH (pg/mL)	261.2 (167.8, 414.6)	258.0 (149.0, 433.0)	0.335
Albumin (g/L)	35.1 ± 5.5	36.5 ± 6.0	0.009
NT-proBNP (pg/ml)	29125.0 (11474.5, 68532.0)	12561.3 (4035.0, 37575.0)	<0.001
Cardiac troponin I (μg/L)	0.044 (0.025, 0.078)	0.037 (0.020, 0.064)	0.009
Creative kinase MB (U/L)	13.8 (10.1, 19.0)	14.0 (10.9, 18.0)	0.471
**Cardiac function**			
NYHA functional classification			
I	0 (0.0%)	110 (44.5%)	<0.001
II	130 (52.6%)	82 (33.2%)	
III	117 (47.4%)	55 (22.3%)	
IV	0 (0.0%)	0 (0.0%)	
**Cardiac structure**			
RVDd (mm)	17.2 ± 3.0	16.8 ± 2.8	0.107
LVPWT (mm)	11.8 ± 2.0	10.8 ± 1.9	<0.001
IVSd (mm)	11.8 ± 2.0	11.2 ± 2.0	<0.001
LVDd (mm)	53.8 ± 6.9	51.2 ± 7.1	<0.001
LAD (mm)	40.5 ± 6.2	37.2 ± 7.2	<0.001
AAO (mm)	33.9 ± 4.1	32.5 ± 4.9	0.001
E/A ratio	0.8 (0.7, 1.3)	0.9 (0.8, 1.3)	0.008
LVEDV (mL)	143.0 (111.5, 174.0)	130.0 (105.0, 163.0)	<0.001
LVESV (mL)	57.0 (43.0, 82.5)	48.0 (38., 74.0)	<0.001
TRVmax (m/s)	2.7 (2.5, 3.2)	2.4 (2.0, 2.8)	<0.001
Septal e’ wave velocity (cm/s)	8.0 ± 0.6	8.2 ± 0.5	0.001
Lateral e’ wave velocity (cm/s)	9.9 ± 0.8	10.2 ± 0.7	<0.001
E/e’	8.3 (6.4, 11.8)	7.2 (6.1, 8.9)	<0.001
LA volume index (mL/m^2^)	37.9 ± 4.2	36.4 ± 4.1	<0.001
PASP (mmHg)	39.0 (30.5, 50.0)	28.0 (21.0, 37.5)	<0.001
PAH [*n* (%)]	145 (58.7%)	71 (28.7%)	<0.001
LVEF (%)	61.4 ± 4.6	60.3 ± 6.7	0.058

Data presented as median (first-third interquartile range) or mean ± SD or number (percentage).

HFpEF, Heart failure with preserved ejection fraction; SBP, systolic blood pressure; DBP, diastolic blood pressure; bpm, beat per minute; TC, total cholesterol; TG, triglycerides; HDL-C, high-density lipoprotein cholesterol; LDL-C, low-density lipoprotein cholesterol; Intact PTH, intact parathyroid hormone; NT-proBNP, N- terminal B- type natriuretic peptide precursor; NYHA functional classification, New York Heart Association Functional Classification; RVDd, right ventricle diastolic diameter; LVPWT, left ventricular posterior wall thickness; AAO, ascending aorta; LVEDV, Left ventricular end-diastolic volume; LVESV, Left ventricular end-systolic volume; E/A ratio, Early-to-late transmitral flow ratio; IVSd, intraventricular septal thickness in diastole; LAD, left atrial diameters; LVDd, left ventricular end-diastolic diameter; TRVmax, maximal tricuspid regurgitation velocity; LA volume index, left atrial volume index; PASP, pulmonary arterial systolic pressure; PAH, pulmonary arterial hypertension; LVEF, left ventricular ejection fraction.

According to the symptoms of heart failure, we graded the cardiac function of patients according to NYHA functional class. Before initiating sacubitril/valsartan, a total of 130 (52.6%) patients were in NYHA functional class II, and 117 (47.4%) were in class III. After treatment with sacubitril/valsartan, the majority of patients had NYHA functional class I (44.5%), and 82 (33.2%) patients had NYHA functional class II. There were only 55 patients that were in NYHA functional class III. There were significant differences in the cardiac function grading before and after the treatment of sacubitril/valsartan among those 247 MHD patients with HFpEF, which suggested that these patients achieved a pronounced improvement in cardiac function.

### Comparison of the echocardiographic outcomes of 247 patients with maintenance hemodialysis before and after initiating sacubitril/valsartan

Based on the echocardiographic findings, we found that after treatment with sacubitril/valsartan, indicators of left ventricular remodeling including LVPWT (11.8 ± 2.0 vs. 10.8 ± 1.9 mm, *P* < 0.001), IVSd (11.8 ± 2.0 vs. 11.2 ± 2.0 mm, *P* < 0.001), LVDd (53.8 ± 6.9 vs. 51.2 ± 7.1 mm, *P* < 0.001), LAD (40.5 ± 6.2 vs. 37.2 ± 7.2 mm, *P* < 0.001), LVEDV [143.0 (111.5, 174.0) vs. 130.0 (105.0, 163.0) ml, *P* < 0.001], and LVESV [57.0 (43.0, 82.5) vs. 48.0 (38.0, 74.0) ml, *P* < 0.001] were significantly reduced. As the parameters of left ventricular diastolic function, E/A ratio [0.8 (0.7, 1.3) vs. 0.9 (0.8, 1.3), *P* = 0.008], TRVmax [2.7 (2.5, 3.2) vs. 2.4 (2.0, 2.8) m/s, *P* < 0.001], septal e’wave velocity (8.0 ± 0.6 vs. 8.2 ± 0.5 cm/s, *P* = 0.001), lateral e’ wave velocity (9.9 ± 0.8 vs. 10.2 ± 0.7 cm/s, *P* < 0.001), E/e’ [8.3 (6.4, 11.8) vs. 7.2 (6.1, 8.9), *P* < 0.001], and LAVI (37.9 ± 4.2 vs. 36.4 ± 4.1 ml/m^2^, *P* < 0.001) were significantly improved by the sacubitril/valsartan. PASP and the proportion of patients with concomitant PAH significantly decreased after the treatment of sacubitril/valsartan. While no significant difference existed in other echocardiographic parameters including RVDd and LVEF (Table 2).

### Safety analysis of sacubitril/valsartan used in those 247 maintenance hemodialysis patients with heart failure with preserved ejection fraction

None of the MHD patients with HFpEF receiving the treatment of sacubitril/valsartan showed severe adverse drug reactions such as hypotension and angioedema. Additionally, there was no significant change in the proportion of patients with hyperkalemia after initiating sacubitril/valsartan (Table 2).

### Comparisons of the clinical data of 211 patients who were already treated with angiotensin converting enzyme inhibitors/angiotensin receptor blockers and 36 patients who were sacubitril/valsartan naive

In this study, 211 patients were already in treatment with ACEi/ARB before being treated with sacubitril/valsartan. The duration of ACEi/ARB therapy in those patients ranged from 7 to 25 months (median: 8.5 months). To analyze whether the results would be affected by the use of ACEi/ARB or not, we provided an analysis to compare the clinical data of 211 patients who were already treated with ACEi/ARB and 36 patients who were sacubitril/valsartan naive.

The results showed that compared with sacubitril/valsartan naive patients, the BMI of those 211 patients who were already treated with ACEi/ARB was heavier (24.1 ± 3.5 vs. 22.3 ± 3.2 kg/m^2^, *P* = 0.035). Besides that, the cause of ESRD was different in the two groups. The proportion of obstructive nephropathy and polycystic kidney was significantly higher in sacubitril/valsartan naive patients (8.3% vs. 0.9%, *P* = 0.023; 13.9% vs. 0.5%, *P* < 0.001). In the use of anti-hypertensive medicine, the proportion of β-blocker and α-blocker was higher in those patients who were already treated with ACEi/ARB (55.6% vs. 78.2%, *P* = 0.006; 27.8% vs. 71.6%, *P* < 0.001) (Table 1). In terms of baseline data, except for the proportion of hyperkalemia (16.7% vs. 3.8%, *P* = 0.002), serum albumin level (38.4 ± 4.9 vs. 34.5 ± 5.3 g/L, *P* < 0.001), and RVDd (18.3 ± 3.0 vs. 17.0 ± 3.0 mm, *P* = 0.023), there were no significant differences between the two groups in terms of cardiac indexes including NT-proBNP and other echocardiographic outcomes (Table 3).

**TABLE 3:** Clinical data at baseline and the end of follow-up of 211 HFpEF patients who were already treated with ACEi/ARB and 36 HFpEF patients who were sacubitril/valsartan naive.

Variables	ACEi/ARB treated patients (*n* = 211)	Sacubitril/Valsartan naive patients (*n* = 36)	*P*-value
**Clinical parameters**			
**SBP (mmHg)**			
Baseline	150.0 ± 23.9	147.6 ± 22.0	0.569
The end of follow-up	137.2 ± 20.2^a^	136.9 ± 24.2^a^	0.935
Percentage change (%)	–7.8 (–20.0, 8.6)	–1.1 (–6.8, 0.5)	0.685
**DBP (mmHg)**			
Baseline	90.7 ± 16.3	87.3 ± 14.5	0.229
The end of follow-up	84.8 ± 14.5^a^	82.9 ± 11.8	0.438
Percentage change (%)	–5.9 (–19.2, 8.7)	–1.2 (–14.4, 8.6)	0.621
**Heart rate (bpm)**			
Baseline	83.4 ± 12.1	83.7 ± 14.1	0.910
The end of follow-up	80.4 ± 8.8^a^	78.5 ± 8.2^a^	0.238
Percentage change (%)	–3.5 (–11.1, 5.0)	–1.3 (–8.3, 4.5)	0.836
**Laboratory values**			
**Urea (mmol/L)**			
Baseline	26.0 (20.1, 33.2)	23.5 (18.3, 29.6)	0.298
The end of follow-up	24.0 (17.1, 32.0)	21.4 (14.6, 25.6)	0.001
Percentage change (%)	–7.9 (–30.7, 20.0)	–18.4 (–37.3, 1.0)	0.130
**Creatinine (μmol/L)**			
Baseline	957.0 (751.5, 1160.1)	957.0 (682.8.0, 1172.0)	0.103
The end of follow-up	764.0 (596.3, 972.0)	732.5 (546.2, 896.3)	0.001
Percentage change (%)	–2.5 (–21.0, 15.0)	–10.0 (–22.7, 6.2)	0.218
**Uric acid (μmol/L)**			
Baseline	387.0 (318.5, 478.3)	359.0 (299.0, 419.5)	0.242
The end of follow-up	356.0 (292.0, 451.0)	295.0 (234.8, 429.8)	0.001
Percentage change (%)	–7.1 (–26.9, 11.0)	–16.8 (–37.8, 10.3)	0.286
**Potassium (mmol/L)**			
Baseline	4.3 ± 0.8	4.7 ± 0.9	0.007
The end of follow-up	4.3 ± 0.8	4.8 ± 0.8	0.003
Percentage change (%)	–0.4 (–11.2,15.9)	3.3 (–12.6, 20.1)	0.684
**Calcium (mmol/L)**			
Baseline	2.1 (1.9, 2.3)	2.1 (2.0, 2.4)	0.641
The end of follow-up	2.2 (2.1, 2.3)	2.2 (2.1, 2.4)	0.269
Percentage change (%)	3.5 (–3.4, 13.4)	4.6 (–3.5, 10.0)	0.957
**Phosphorus (mmol/L)**			
Baseline	2.0 (1.6, 2.5)	1.8 (1.3, 2.2)	0.114
The end of follow-up	1.9 (1.5, 2.3)	1.8 (1.5, 2.4)	0.619
Percentage change (%)	–1.8 (–26.2, 21.1)	22.3 (–20.3, 35.0)	0.743
**Hyperkalemia [n (%)]**			
Baseline	8 (3.8%)	6 (16.7%)	0.002
The end of follow-up	13 (6.2%)	7 (19.4%)	0.007
**Intact PTH (pg/mL)**			
Baseline	276.0 (170.0, 417.8)	251.5 (151.7, 343.3)	0.332
The end of follow-up	266.0 (125.3, 426.5)	199.0 (130.0, 359.0)	0.328
Percentage change (%)	–2.5 (–45.2, 45.5)	–4.5 (–45.3, 25.2)	0.625
**Albumin (g/L)**			
Baseline	34.5 ± 5.3	38.4 ± 4.9	< 0.001
The end of follow-up	35.6 ± 5.8^a^	41.3 ± 4.6^a^	< 0.001
Percentage change (%)	2.6 (–6.1, 13.5)	8.1 (–0.2, 18.5)	0.108
**NT-proBNP (pg/ml)**			
Baseline	32555.0 (9165.5, 82722.5)	18489.7 (10544.9, 34141.7)	0.878
The end of follow-up	9316.9 (3617.2, 30715.2)^a^	10537.2 (2541.8, 29280.2)^a^	0.757
Percentage change (%)	–35.8 (–85.9, 38.9)	–70.8 (–83.5, –9.3)	0.700
**Cardiac troponin I (μg/L)**			
Baseline	0.049 (0.025, 0.072)	0.035 (0.023, 0.045)	0.375
The end of follow-up	0.043 (0.022, 0.065)^a^	0.031 (0.021, 0.105)	0.767
Percentage change (%)	–9.8 (–63.6, 55.7)	–7.7 (–38.9, 76.1)	0.555
**Creative kinase MB (U/L)**			
Baseline	13.6 (10.9, 18.7)	12.0 (9.3, 17.0)	0.655
The end of follow-up	14.3 (10.8, 18.3)	16.8 (11.4, 20.3)	0.649
Percentage change (%)	2.2 (–27.5, 33.0)	13.8 (–15.8, 48.3)	0.135
**Cardiac structure**			
**RVDd (mm)**			
Baseline	17.0 ± 3.0	18.3 ± 3.0	0.023
The end of follow-up	16.6 ± 2.8	17.5 ± 2.7	0.098
Percentage change (%)	0.0 (–15.8, 5.9)	–17.0 (–19.0, 0.0)	0.765
**LVPWT (mm)**			
Baseline	11.7 ± 1.9	11.7 ± 2.0	0.967
The end of follow-up	10.8 ± 1.9^a^	10.6 ± 1.5^a^	0.432
Percentage change (%)	–8.3 (–21.1, 3.8)	–3.8 (–28.9, 9.8)	0.760
**IVSd (mm)**			
Baseline	11.8 ± 1.9	11.9 ± 1.9	0.774
The end of follow-up	11.2 ± 1.9^a^	11.0 ± 1.7^a^	0.536
Percentage change (%)	0.0 (–16.7, 7.7)	0.0 (–25.0, 10.0)	0.983
**LVDd (mm)**			
Baseline	53.8 ± 7.0	53.7 ± 5.7	0.928
The end of follow-up	51.2 ± 7.2^a^	51.1 ± 6.4	0.924
Percentage change (%)	–3.7 (–11.9, 2.0)	–8.2 (–15.6, –0.51)	0.991
**LAD (mm)**			
Baseline	40.3 ± 6.2	40.9 ± 6.4	0.583
The end of follow-up	37.3 ± 6.9^a^	37.2 ± 8.0^a^	0.933
Percentage change (%)	–7.3 (–19.1, 5.0)	–18.5 (–27.6, –8.8)	0.419
**AAO (mm)**			
Baseline	33.9 ± 4.2	33.6 ± 3.7	0.692
The end of follow-up	32.5 ± 4.9^a^	32.0 ± 3.8	0.508
Percentage change (%)	–2.5 (–13.5, 10.0)	–2.9 (–18.1, 2.8)	0.463
**E/A Ratio**			
Baseline	0.9 (0.7, 1.3)	1.3 (0.8, 1.7)	0.175
The end of follow-up	0.8 (0.7, 1.3)^a^	0.8 (0.8, 1.3)	0.894
Percentage change (%)	–3.9 (–33.5, 21.8)	–10.0 (–43.3, 5.1)	0.320
**LVEDV (mL)**			
Baseline	148.0 (111.0, 186.5)	136.0 (122.0, 193.0)	0.959
The end of follow-up	129.0 (105.0, 167.0)^a^	128.0 (94.0, 158.5)^a^	0.586
Percentage change (%)	–3.8 (–25.6, 10.7)	–19.0 (–35.3, –1.2)	0.531
**LVESV (mL)**			
Baseline	63.0 (44.0, 87.5)	52.0 (46.0, 70.0)	0.755
The end of follow-up	48.0 (38.0, 75.0)^a^	51.0 (34.5, 75.5)^a^	0.589
Percentage change (%)	–9.2 (–32.2, 18.2)	–12.6 (–52.7, 17.7)	0.589
**TRVmax (m/s)**			
Baseline	2.9 (2.5, 3.2)	2.7 (2.5, 3.0)	0.245
The end of follow-up	2.3 (2.0, 2.8)^a^	2.4 (2.0, 3.0)^a^	0.364
Percentage change (%)	–15.4 (–25.9, 0.0)	–17.7 (–27.4, –7.9)	0.838
**Septal e’ wave velocity (cm/s)**			
Baseline	8.0 ± 0.6	7.8 ± 0.7	0.058
The end of follow-up	8.3 ± 0.5^a^	8.2 ± 0.5	0.713
Percentage change (%)	0.9 (–2.8, 8.6)	1.4 (0.0, 17.2)	0.073
**Lateral e’ wave velocity (cm/s)**			
Baseline	9.9 ± 0.8	9.7 ± 0.9	0.162
The end of follow-up	10.0 ± 0.7^a^	10.3 ± 0.6^a^	0.343
Percentage change (%)	0.9 (–2.8, 8.6)	1.4 (0.0, 17.2)	0.071
**E/e’**			
Baseline	8.2 (6.4, 11.6)	10.1 (6.5, 12.9)	0.101
The end of follow-up	7.2 (6.2, 9.0)^a^	7.2 (6.1, 8.8)^a^	0.558
Percentage change (%)	–12.1 (–32.9, 17.3)	–33.5 (–53.3, –15.4)	0.027
**LA volume index (mL/m^2^)**			
Baseline	37.9 ± 4.2	37.7 ± 3.9	0.791
The end of follow-up	36.4 ± 4.1^a^	36.4 ± 4.2	0.995
Percentage change (%)	–2.8 (–11.1, 2.7)	0.0 (–16.3, 0.0)	0.968
**PASP (mmHg)**			
Baseline	41.0 (32.0, 50.0)	35.0 (31.0, 40.0)	0.054
The end of follow-up	28.0 (21.0, 41.0)^a^	28.0 (21.0, 42.5)^a^	0.235
Percentage change (%)	–25.0 (–46.9, –6.8)	–21.2 (–34.6, –11.0)	0.339
**PAH [*n* (%)]**			
Baseline	119 (56.4%)	26 (72.2%)	0.075
The end of follow-up	58 (27.5%)^a^	13 (36.1%)^a^	0.291
**Percentage change (%)**			
**LVEF (%)**			
Baseline	59.6 ± 6.3	56.8 ± 5.4	0.510
The end of follow-up	59.8 ± 5.7	58.9 ± 7.9	0.668
Percentage change (%)	0.0 (–4.5, 4.5)	2.4 (–1.9, 6.9)	0.200

Data presented as median (first-third interquartile range) or mean ± SD or number (percentage). Percentage change = (Data at the end of follow up- Data at baseline) × 100/Data at baseline.

^*a*^Stands for *p* < 0.05 vs. baseline.

HFpEF, Heart failure with preserved ejection fraction; ACEi, angiotensin- converting enzyme inhibitors; ARB, angiotensin II receptor blocker; SBP, systolic blood pressure; DBP, diastolic blood pressure; bpm, beat per minute; Intact PTH, intact parathyroid hormone; NT-proBNP, N- terminal B- type natriuretic peptide precursor; RVDd, right ventricle diastolic diameter; LVPWT, left ventricular posterior wall thickness; AAO, ascending aorta; LVEDV, Left ventricular end-diastolic volume; LVESV, Left ventricular end-systolic volume; E/A ratio, Early-to-late transmitral flow ratio; IVSd, intraventricular septal thickness in diastole; LAD, left atrial diameters; LVDd, left ventricular end-diastolic diameter; TRVmax, maximal tricuspid regurgitation velocity; LA volume index, left atrial volume index; PASP, pulmonary arterial systolic pressure; PAH, pulmonary arterial hypertension; LVEF, left ventricular ejection fraction.

The cardiac function of those 211 patients who were already treated with ACEi/ARB was improved which was demonstrated by the improvement of NT-proBNP, TNI, LVPWT, IVSd, LVDd, LAD, LVEDV, LVESV, E/A ratio, TRVmax, septal e’ wave velocity, lateral e’ wave velocity, E/e’, and LA volume index. Similar results were observed in 36 patients who were sacubitril/valsartan naive (Table 3).

In order to analyze the differences in the effect of sacubitril/valsartan on these two groups, we compared the differences in the percentage change in relevant indicators between the two groups. As shown in Table 3, in addition to E/e’ [–33.5 (–53.3, –15.4) vs. –12.1 (–32.9, 17.3), *P* = 0.027], there were no significant differences in percentage change in clinical parameters, laboratory values, and other echocardiographic outcomes between the two groups (Table 3).

## Discussion

To the best of our knowledge, this is the first report on the treatment of sacubitril/valsartan in MHD patients with HFpEF. With the advance of hemodialysis technology, the survival of patients with ESRD is progressively prolonged (21). However, the mortality and morbidity of patients with MHD remain high (22). Heart failure is one of the most common co-morbidities in patients with dialysis, which is also the main cause of mortality and morbidity among patients with MHD (23). HFpEF is the most common form of heart failure in patients with MHD (7). In patients with MHD, both traditional risk factors of HFpEF including hypertension and diabetes as well as non-traditional risk factors, such as reduced renal function, increased volume load, the renin–angiotensin–aldosterone system (RAAS) activation, anemia, calcium, and phosphate metabolism disorders, and arteriovenous fistulas can also contribute to cardiac hypertrophy and ventricular remodeling (24). Heart failure, in turn, can reduce renal perfusion, thereby activating the RAAS, aggravating heart failure, and forming a vicious cycle, in which the natriuretic peptide system and the RAAS play an important role (25). The aim of the treatment of MHD with HFpEF is to preserve residual renal function and reduce cardiovascular events.

Compared with heart failure with reduced ejection fraction (HFrEF), pharmacological therapies for HFpEF including ACEi, ARB, and beta-blocker have generally been disappointing and show no convincing evidence of mortality or morbidity reduction (9–13). Therefore, how to break the dilemma of the treatment of HFpEF in patients with MHD is an urgent problem at present. Sacubitril/valsartan is an angiotensin-receptor neprilysin inhibitor. As a receptor antagonist of angiotensin II, sacubitril/valsartan can inhibit RAAS activation, vasoconstriction, and cardiomyocyte fibrosis. The inhibition of neprilysin blocks natriuretic peptide hydrolysis, enhances the activity of the natriuretic peptide system, dilates blood vessels, expels natriuresis, and reduces cardiac workload (26). In patients with anuric MHD, sacubitril/valsartan can still exert vasodilatory and relieve cardiac remodeling directly. In addition, its plasma protein binding is high and it should not be cleared by hemodialysis (27).

PARAGON-HF is a trial published in 2019 evaluating the efficacy and safety of sacubitril/valsartan vs. valsartan in patients with HFpEF (28). Although the study showed that sacubitril/valsartan did not significantly reduce the composite endpoints of death and total heart failure hospitalization in patients with HFpEF, the primary endpoint was significantly reduced in the low LVEF group and female group in the prespecified subgroup analysis. PARAGON-HF trial *post hoc* analysis showed that serum levels of NT-proBNP and cardiac troponin T were significantly lower in the sacubitril/valsartan group compared with the valsartan group, suggesting that sacubitril/valsartan better reduces the hemodynamic loading of the ventricles and protects against cardiomyocyte damage. This is also an important mechanism of sacubitril/valsartan in the treatment of HFpEF. The PARALLAX trial, published in 2020, reported a significant 16.4% reduction of NT-proBNP in patients treated with sacubitril/valsartan compared with patients treated with optimal individualized therapy after the treatment of 12 weeks, and this difference was maintained until the end of follow-up. The largest highlight of the study was the significant reduction in the risk of first heart failure hospitalization by 51% and the risk of a composite event of HF hospitalization or death by 36% in the sacubitril/valsartan group compared with the control group (29). The results provide evidence for the use of sacubitril/valsartan in HFpEF.

At present, there is still no relevant research on whether sacubitril/valsartan can effectively treat HFpEF among patients with MHD. Our findings showed that sacubitril/valsartan significantly improved and stabilized cardiac function in MHD patients with HFpEF, which was supported by clinical and laboratory parameters, including relief of heart failure signs and symptoms, decreased NT-proBNP levels, TNI, and heart rate, and improvement of echocardiographic outcomes.

NT-proBNP is the most prominent biological marker for the evaluation of heart failure (30). Our study showed that NT-proBNP levels were significantly decreased after initiating sacubitril/valsartan in patients with MHD, which is consistent with the findings of the PARALLAX trial. Peripheral blood cardiac troponin is a marker of myocardial injury and is important for risk stratification and prognostic assessment in HFpEF (30, 31). The PARAGON-HF trial showed that sacubitril/valsartan significantly reduced high-sensitivity cardiac troponin T (hs-TnT) levels compared with valsartan, and hs-TnT may be helpful in identifying patients with HFpEF who are more likely to benefit from sacubitril/valsartan (32). Our study showed that cardiac troponin levels were significantly reduced by sacubitril/valsartan in MHD patients with HFpEF, which is consistent with the findings of the PARAGON-HF trial.

Left ventricular remodeling is another central feature in the pathophysiology of HFpEF (33). The incidence of left ventricular hypertrophy in patients with hemodialysis is as high as 75% (34). A meta-analysis showed that sacubitril/valsartan could improve left ventricular mass index and left atrial volume in patients with HFpEF (35). Sacubitril/valsartan can reduce neprilysin activity and increase natriuretic peptide concentration, further reduce the heart load, delay the occurrence of myocardial fibrosis, and relax blood vessels. ARBs in sacubitril/valsartan not only reduced aldosterone levels but also partially counteracted the increase of angiotensin II levels induced by neprilysin inhibitor. In addition, sacubitril/valsartan can obviously increase cGMP levels in circulation, inhibit the expression of gene programs related to cardiac fibroblasts, reduce the activation and proliferation of fibroblasts, improve the degree of myocardial stiffness, and delay cardiac hypertrophy and remodeling (36, 37). Our studies showed that LAD, LVPWT, IVSd, LVDd, LVEDV, and LVESV were significantly decreased after the treatment of sacubitril/valsartan, which suggested that sacubitril/valsartan could reverse left ventricular remodeling to some extent, which is consistent with previous trials among patients with HFrEF (38–41). However, our study showed no significant difference in structural changes in the right ventricular after the treatment of sacubitril/valsartan among the MHD patient with HFpEF, which might suggest that structural changes in the right ventricular require a longer treatment period.

Left ventricular diastolic dysfunction is the major pathological mechanism of HFpEF. In echocardiography, some parameters including Mitral E wave velocity, DT of E wave, Septal e’ wave velocity, Lateral e’ wave velocity, E/e’, LAVI, TRVmax, pulmonary vein AR wave duration, and pulmonary vein flow with S/D waves ratio can reflect left ventricular diastolic function. Previous studies showed that the E/A ratio could be improved with the treatment of sacubitril/valsartan in patients with HFrEF (42). In our study, we found that cardiac diastolic function showed a trend of improvement after the treatment with sacubitril/valsartan, demonstrated by the improvement of E/A ratio, TRVmax, septal e’ wave velocity, lateral e’ wave velocity, E/e’, and LAVI. In addition, we also found that sacubitril/valsartan therapy decreased the PASP in MHD patients with HFpEF, which may be related to the improvement of left heart function. Meanwhile, previous studies confirmed that neprilysin inhibition by sacubitril could relax the pulmonary arteries, inhibit pulmonary artery smooth muscle cell proliferation, decrease the metabolism of vasoconstrictors, and reduce pulmonary vascular resistance, which might be the underlying mechanism of the reduction of PASP (43). The findings will provide a new idea for the treatment of MHD patients with concomitant PAH.

This manuscript has certain limitations. First, this is a retrospective study, whose level of evidence is lower than that of randomized controlled trials, which may affect the reliability of the conclusions. Therefore, randomized controlled trials with large sample sizes are needed to confirm our findings, and longer follow-ups will be needed to assess the long-term efficacy and safety of sacubitril/valsartan. Second, the absence of a proper control group is also one major shortcoming of this study, and it is impossible to be sure whether sacubitril/valsartan is superior to other therapeutic regimens including general treatment. Third, we made the diagnosis of HFpEF according to the ESC guidelines. However, we found that the proportion of patients with a low H2FPEF score was higher, which makes the diagnosis of HFpEF in this manuscript seem to be exaggerated. Of the 247 patients with MHD, 69 patients had an H2FPEF score of 1, 82 patients had a score of 2, 69 patients had a score of 3, 16 patients had a score of 4, 2 patients had a score of 5, 7 patients had a score of 6, 1 patient had a score of 7, and 1 patient had a score of 8. Besides that, this study enrolled 247 patients with HFpEF undergoing hemodialysis with 45.8 y/o and a BMI of 23.4 kg/m^2^. These patients are different from other international HFpEF trials, including PARAGON-HF (72.7 y/o, BMI 30.2 kg/m^2^), EMPEROR-PRESERVED (71.8 y/o BMI 29.8 kg/m^2^), and TOPCAT (age 68.7 y/o, BMI 31 kg/m^2^). We reviewed the studies on HFpEF among patients with ESRD. Those studies enrolled ESRD patients with HFpEF with 59.5 y/o and BMI 27.1 kg/m^2^ (7), 54 y/o and BMI 23.5 kg/m^2^ (44), and 60.4 y/o, and BMI 23.4 kg/m^2^ (45). From these above studies, we found that compared with patients without ESRD, HFpEF patients with ESRD appeared to have some different characteristics, such as age at onset, BMI, and HF2PEF score. There may be several factors contributing to this phenomenon. Previous studies showed a high prevalence of HFpEF in patients with MHD (7), and several renal factors including the activation of the RAAS, anemia, hyperphosphatemia, increased levels of FGF-23, and uremic toxins have impact on the HFpEF (46), which lead to HFpEF being more prevalent in patients with MHD. Owing to the uniqueness of ESRD, the characteristics of MHD patients with HFpEF are different from those of patients without renal disease. In the H2FPEF scoring system, only the common risk factors of HFpEF are highlighted, and the characteristics of ESRD are not considered. It is unclear whether it is appropriate to use the H2FPEF scoring system to diagnose HFpEF in patients with MHD, which may need to be confirmed in further studies. Fourth, in clinical practice, complete echocardiographic indicators reflecting diastolic function were not collected in this study. Thus, future study is needed to obtain complete clinical data.

## Conclusion

This is the first study about sacubitril/valsartan treatment for HFpEF among patients with MHD and the results showed the effectiveness and safety of sacubitril/valsartan in MHD patients with HFpEF, which will bring hope for these patients.

## Data availability statement

The raw data supporting the conclusions of this article will be made available by the authors, without undue reservation.

## Ethics statement

The studies involving human participants were reviewed and approved by the Ethics Committee of The First Affiliated Hospital of Zhengzhou University. The patients/participants provided their written informed consent to participate in this study.

## Author contributions

YG, LT, and PL: study design and manuscript preparation. TP, XL, TW, YW, LY, MR, and LW: literature search, data collection, statistical analysis, and data interpretation. All authors contributed to the article and approved the submitted version.
