# Supplementary material for: Effects of sacubitril/valsartan in ESRD patients undergoing hemodialysis with HFpEF
Source: Front Cardiovasc Med. 2022 Nov 9;9:955780. doi: 10.3389/fcvm.2022.955780 (PMC9681904; doi:10.3389/fcvm.2022.955780)
Supplement: Supplementary file 1 [file Table_1.docx]

| Supplement table 1 \| Comparison of clinical data of 211 MHD patients without heart failure and 247 MHD patients with HFpEF. | | | |
| --- | --- | --- | --- |
| **Variables** | **MHD patients without heart failure (n=211)** | **MHD patients with HFpEF (n=247)** | ***P*-valve** |
| NT-proBNP (pg/ml) | 2357.0 (1167.2, 4968.0) | 29125.0 (11474.5, 68532.0) |  |
| **CARDIAC STRUCTURE** |  |  |  |
| RVDd (mm) | 15.8±1.2 | 17.2±3.0 | 0.001 |
| LVPWT (mm) | 9.8±1.5 | 11.8±2.0 | <0.001 |
| IVSd (mm) | 9.9±1.7 | 11.8±2.0 | <0.001 |
| LVDd (mm) | 48.9±6.0 | 53.8±6.9 | <0.001 |
| LAD (mm) | 35.4±5.1 | 40.5±6.2 | <0.001 |
| AAO (mm) | 31.7±3.2 | 33.9±4.1 | <0.001 |
| E/A Ratio | 0.8 (0.7, 1.3) | 0.8 (0.7, 1.3) | 0.021 |
| LVEDV (mL) | 100.0 (95.0, 144.0) | 143.0 (111.5, 174.0) | <0.001 |
| LVESV (mL) | 37.0 (35.0, 54.0) | 57.0 (43.0, 82.5) | <0.001 |
| TRVmax (m/s) | 2.2±0.4 | 2.7 (2.5, 3.2) | <0.001 |
| Septal e’ wave velocity (cm/s) | 8.3±0.6 | 8.0±0.6 | 0.001 |
| Lateral e′ wave velocity (cm/s) | 10.3±0.5 | 9.9±0.8 | 0.002 |
| E/e′ | 7.2±1.4 | 8.3 (6.4, 11.8) | 0.001 |
| LA volume index (mL/m^2^) | 33.8±1.7 | 37.9±4.2 | <0.001 |
| PASP (mmHg) | 25.6±6.2 | 39.0 (30.5, 50.0) | <0.001 |
| LVEF (%) | 63.1±2.8 | 61.4±4.6 | <0.001 |
| Data presented as median (first-third interquartile range) or mean ± SD. MHD, maintenance hemodialysis; HFpEF, Heart failure with preserved ejection fraction; NT-proBNP, N- terminal B- type natriuretic peptide precursor; RVDd, right ventricle diastolic diameter; LVPWT, left ventricular posterior wall thickness; AAO, ascending aorta; LVEDV, Left ventricular end-diastolic volume; LVESV, Left ventricular end-systolic volume; E/A raito, Early-to-late transmitral flow ratio; IVSd, intraventricular septal thickness in diastole; LAD, left atrial diameters; LVDd, left ventricular end-diastolic diameter; TRVmax, maximal tricuspid regurgitation velocity; LA volume index, left artial volume index; PASP, pulmonary arterial systolic pressure; LVEF, left ventricular ejection fraction. | | | |
